# Longitudinal study of risk factors for decreased cross-sectional area of psoas major and paraspinal muscle in 1849 individuals

**DOI:** 10.1038/s41598-021-96448-8

**Published:** 2021-08-20

**Authors:** Yoichi Murata, Eiichiro Nakamura, Manabu Tsukamoto, Toru Nakagawa, Masaru Takeda, Mio Kozuma, Takayuki Kadomura, Kenichiro Narusawa, Kenji Shimizu, Soshi Uchida, Takeshi Hayashi, Akinori Sakai

**Affiliations:** 1 Department of Orthopaedic Surgery, University of Occupational and Environmental Health, 1-1 Iseigaoka, Yahatanishiku, Kitakyushu, Fukuoka 8078555 Japan; 2 Occupational Health Section, Hitachi Health Care Center, Hitachi Ltd., 4-3-16 Osecho, Hitachi, Ibaraki 3170076 Japan; 3 University of Occupational and Environmental Health, 1-1 Iseigaoka, Yahatanishiku, Kitakyushu, Fukuoka 8078555 Japan; 4 Health Care Business Unit, Hitachi Ltd., 2 Shintoyofuta, Kashiwa, Chiba 2770804 Japan; 5 Department of Orthopaedic Surgery, Nakashibetsu Town Hospital, 9-1-1, 10-jo Minami, Nakashibetsu-cho Nishi, Hokkaido 0861110 Japan; 6 Department of Orthopaedic Surgery, Tobata Kyoritsu Hospital, 2-5-1 Sawami, Tobata, Kitakyushu, Fukuoka 8040093 Japan; 7 Department of Orthopaedic Surgery and Sports Medicine, Wakamatsu Hospital of University of Occupational and Environmental Health, 1-17-1 Hamamachi, Wakamatsu, Kitakyushu, Fukuoka 8080024 Japan

**Keywords:** Orthopaedics, Epidemiology

## Abstract

This 10-year retrospective observational study investigated longitudinal losses in psoas major and paraspinal muscle area in 1849 healthy individuals (1690 male, 159 female) screened using computed tomography. Logistic regression analysis revealed significant decreases in psoas major and paraspinal muscle area at 10 years relative to the baseline area regardless of age or sex, starting at 30 years of age. Only aging [≥ 50 s (odds ratio [OR]: 1.72; 95% confidence interval [CI] 1.05–2.84; *p* = 0.03) and ≥ 60 s (OR: 2.67; 95% CI 1.55–4.60; *p* < 0.001)] was a risk factor for decreases in psoas major area. Age ≥ 60 years (OR: 2.05; 95% CI 1.24–3.39; *p* = 0.005), body mass index ≥ 25 kg/m^2^ (OR: 1.32; 95% CI 1.01–1.73; *p* = 0.04), and visceral fat ≥ 100 cm^2^ (OR: 1.61; 95% CI 1.20–2.15; *p* = 0.001) were risk factors for decreases in paraspinal muscle area. Physical activity ≥ 900 kcal/week (OR: 0.68; 95% CI 0.50–0.94; *p* = 0.02) attenuated paraspinal muscle area loss in male. Our study demonstrated that walking > 45 min daily (Calories = METs (walking: 3.0) × duration of time (h) × weight (60 kg) × 1.05) can reduce paraspinal muscle loss, which may in turn decrease the risk of falls, low-back pain, and sarcopenia.

## Introduction

Sarcopenia is characterized by the progressive loss of skeletal muscle mass and strength and presents a risk for adverse outcomes, including physical disability, poor quality of life (QOL), and death^1,2^. This condition increases the risk of falls in older individuals, often resulting in fractures in areas such as the femoral neck^3–5^. These fractures negatively affect QOL and increase the number of bedridden patients and medical costs^6,7^. Tinetti et al.^8^ reported that walking disability and the risk of falls would be caused by the decrease of paraspinal muscle strength. Indeed, several studies have reported an association between paraspinal muscle size or morphology and low back pain. Ranger et al.^9^ longitudinally investigated the relationship between paraspinal muscle cross-sectional area and both the intensity of low back pain and related disability at 12-months. The authors concluded that paraspinal muscle cross-sectional area could predict disability from low back pain but not pain intensity. A systematic review regarding the association between paraspinal muscle cross-sectional area and low back pain reported that the area of the multifidus muscle—but not of the erector spinae, psoas, or quadratus lumborum—was negatively associated with the risk of low back pain^10^. Thus, preventing the decrease of paraspinal muscle strength is important for reducing adverse events, especially in countries with rapidly aging populations.

Current research shows an association between skeletal muscle area and specific characteristics. Landi et al. found that cerebrovascular disease and osteoarthritis were specific risk factors of sarcopenia in older patients^11^. Raval et al. determined that higher body mass index (BMI) in individuals with peripheral artery disease was associated with more adverse changes in calf muscle characteristics in a 2-year longitudinal study^12^.

Few longitudinal studies in healthy populations have reported psoas major and paraspinal muscle changes except for a small survey or the young generation, despite losses being associated with falls and back pain^13,14^. Therefore, this study aimed to longitudinally compare psoas major and paraspinal muscle area in large number of healthy individuals and determine risk factors associated with given changes. We hypothesized that risk factors, such as obesity and lack of exercise, would cause psoas major and paraspinal muscle area loss, starting in individuals in their 40 s.

## Results

### Individuals

**Figure 1:**
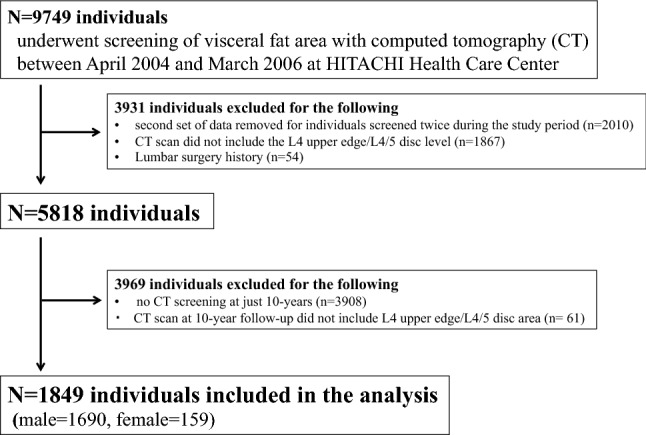
Study flow diagram.

Of 9749 individuals, 3931 were excluded based on preliminary criteria. Specifically, 2010 participants underwent computed tomography (CT) screenings twice from 2004 to 2006, and the second set of data was excluded. In 1867 individuals, cross-sectional CT did not include the area between the L4 upper edge and L4/5 disc. Individuals with a history of lumbar surgery were excluded (n = 54). Individuals who did not undergo a CT screening of visceral fat at 10 years and those with a CT scan that did not measure the area between the L4 upper edge and L4/5 disc 10 years after their initial screening (n = 3908 and n = 61, respectively) were also excluded. In total, 1849 individuals (1690 male, 159 female) met the inclusion criteria. The recruitment process and application of the exclusion criteria are described in Fig. 1.

### Baseline demographic variables

Demographic data according to age groups are summarized in Table 1. No significant differences in BMI were observed between age groups for both sexes, while body fat and subcutaneous fat in male participants decreased with age. Conversely, despite no change in BMI, visceral fat area increased until participants were in their 50 s for both sexes, most notably in male participants in their 30 s and 40 s. Physical activity in male participants increased with age.

**Table 1:** Summary demographic and health characteristics of the individuals at baseline.

	Male (n = 1690)						Female (n = 159)				
	30 s (n = 135)	40 s (n = 424)	50 s (n = 855)	60 s (n = 276)	F	*p* value	40 s (n = 27)	50 s (n = 101)	60 s (n = 31)	F	*p* value
BMI (kg/m^2^)	24.4 ± 3.4 (17.9–35.8)	24.6 ± 3.2 (17.1–36.1)	23.9 ± 2.6 (16.7–33.4)	23.7 ± 2.3 (17.6–31.7)	1.60	0.188	23.0 ± 3.5 (16.5–32.4)	23.3 ± 3.0 (18.3–33.0)	22.2 ± 2.0 (19.2–28.0)	1.93	0.149
Body fat (%)	23.4 ± 4.7 (12.1–37.2)	23.1 ± 4.9 (0–39.2)	21.9 ± 4.1 (10.0–38.6)	21.2 ± 4.2 (10.0–32.2)	14.97	< 0.001	28.4 ± 5.8 (17.5–39.1)	29.1 ± 5.5 (16.2–44.5)	27.6 ± 4.2 (19.7–35.2)	1.44	0.240
Visceral fat (cm^2^)	108.3 ± 54.0 (10.8–244.0)	127.9 ± 52.0 (9.6–302.1)	131.8 ± 54.2 (2.9–317.7)	128.2 ± 53.6 (0–279.0)	7.55	< 0.001	62.6 ± 36.9 (13.8–151.0)	87.9 ± 49.2 (0–227.0)	81.9 ± 34.3 (20.0–140.0)	3.20	0.044
Subcutaneous fat (cm^2^)	146.6 ± 76.5 (15.2–417.0)	143.3 ± 61.5 (11.0–402.8)	124.9 ± 45.6 (7.0–272.1)	113.8 ± 40.5 (18.5–260.0)	25.17	< 0.001	155.6 ± 72.1 (61.7–317.0)	183.1 ± 73.3 (33.0–381.0)	162.4 ± 48.8 (61.0–278.0)	2.65	0.074
Physical activity (kcal/week)	501.8 ± 393.6 (0–1950.0)	606.8 ± 514.4 (0–4620.0)	760.2 ± 1,025.9 (0–14,375.0)	960.5 ± 1,220.0 (0–9562.5)	11.27	< 0.001	610.4 ± 962.7 (0–4800.0)	537.6 ± 975.2 (0–4890.0)	697.6 ± 958.4 (0–3750.0)	0.49	0.612
Smoker (%)	85 (63.0)	243 (57.3)	387 (45.3)	85 (30.8)	–	–	4 (14.3)	3 (2.9)	1 (2.9)	–	–
Drinker (%)	103 (76.3)	338 (79.7)	665 (77.8)	202 (73.2)	–	–	10 (35.7)	21 (20.6)	4 (11.4)	–	–
Area of psoas major (cm^2^)	29.2 ± 4.7 (14.7–44.0)	28.7 ± 5.1 (15.2–45.8)	25.7 ± 4.7 (9.3–43.1)	24.2 ± 4.3 (12.5–39.7)	74.85	< 0.001	17.3 ± 3.5 (10.0–26.8)	14.7 ± 3.3 (7.4–23.7)	14.0 ± 2.5 (8.0–18.6)	8.10	< 0.001
Fat area of psoas major (cm^2^)	0.8 ± 0.6 (0.1–3.1)	1.0 ± 0.6 (0–3.1)	0.9 ± 0.6 (0–4.8)	1.0 ± 0.7 (0–3.3)	1.99	0.114	0.7 ± 0.6 (0.2–2.4)	0.8 ± 0.6 (0–3.1)	0.7 ± 0.6 (0.1–2.5)	0.55	0.580
Area of paraspinal muscle (cm^2^)	50.2 ± 7.7 (33.2–78.0)	48.0 ± 7.2 (29.5–71.9)	46.1 ± 6.6 (18.4–69.8)	44.8 ± 6.5 (28.4–63.7)	26.15	< 0.001	38.1 ± 5.6 (28.6–52.8)	35.6 ± 4.8 (23.4–47.6)	35.0 ± 5.1 (21.2–44.8)	2.59	0.078
Fat area of paraspinal muscle (cm^2^)	1.7 ± 1.3 (0.1–10.2)	1.9 ± 1.3 (0.1–11.3)	2.2 ± 1.5 (0–11.4)	2.7 ± 1.9 (0–11.2)	20.12	< 0.001	2.5 ± 1.4 (0.4–5.6)	3.6 ± 2.2 (0.2–9.2)	3.8 ± 2.5 (0.7–13.2)	3.40	0.036
Fat rate of psoas major area (%)	2.9 ± 1.9 (0.2–9.8)	3.3 ± 2.1 (0.1–11.6)	3.7 ± 2.4 (0–26.6)	4.2 ± 2.8 (0–12.7)	11.55	< 0.001	4.3 ± 3.0 (1.1–12.5)	5.6 ± 4.0 (0.2–24.2)	5.4 ± 5.0 (0.9–24.9)	1.28	0.282
Fat rate of paraspinal muscle area (%)	3.3 ± 2.6 (0.2–18.2)	3.9 ± 2.7 (0.2–21.7)	4.8 ± 3.5 (0.1–25.3)	6.0 ± 4.6 (0.1–26.5)	28.49	< 0.001	6.7 ± 3.9 (1.1–15.9)	10.4 ± 6.8 (0.6–29.6)	11.4 ± 9.1 (0.7–13.2)	3.78	0.025

Data are expressed as mean ± standard deviation (range) except smoker and drinker.

*BMI* body mass index.

### Baseline muscle, fat area composition, and fat rate of muscles

Baseline psoas major and paraspinal muscle composition (total for the right and left sides together) for each age group is presented in Table 1. Areas of both the psoas major and the paraspinal muscles decreased with age in male participants. Although no significant differences in the fat area of the psoas major were observed among age groups, paraspinal muscle fat area increased with age in both sexes.

### Changes in the muscle area between baseline and 10 years

**Figure 2:**
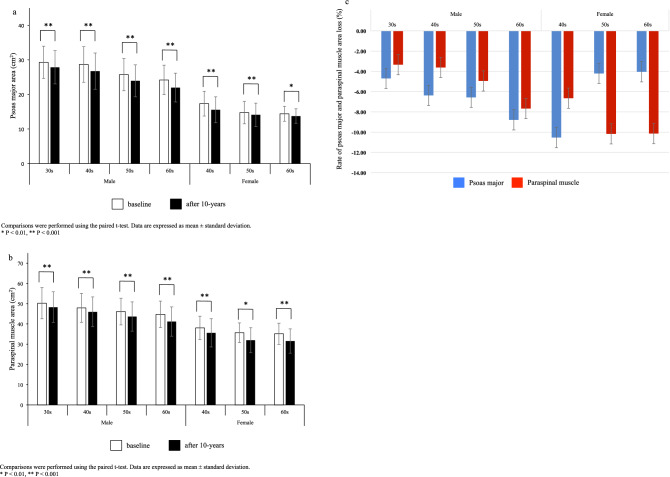
Differences in psoas major and paraspinal muscle areas over a 10-year period. (**a**) Differences in psoas major muscle area over 10 years. (**b**) Differences in paraspinal muscle area over 10 years. (**c**) Rate of psoas major and paraspinal muscle areas loss over 10 years.

The 10-year changes in psoas major and paraspinal muscle area by age group and sex are shown in Fig. 2a,b. At the 10-year follow up, psoas major and paraspinal muscle areas were significantly smaller than those at baseline regardless of age or sex. For individuals in the 30–39-year age group, significant area loss was already present in the psoas major and paraspinal muscle. The rate of both the psoas major and paraspinal muscle area loss were 4–6% per decade for male participants aged 30–59 years and approximately 8% for male participants in their 60 s. The rate of paraspinal muscle area loss was approximately 6% for female participants in their 40 s and approximately 10% per decade for female participants in their 50 s and 60 s. In contrast, the highest reduction of the psoas muscle area was in female participants aged 40–49 years. Although male participants in their 50 s and 60 s had larger psoas major area reduction rates than female participants of the same age, female participants in their 50 s and 60 s had the largest paraspinal muscle area loss (Fig. 2c).

### Multivariate analysis

The mean loss in the highest quartile of psoas major area was − 20.0 ± 6.2% (range − 45.1 to − 13.1%) (n = 423), and paraspinal muscle area loss was − 19.6 ± 9.7% (range − 67.1 to − 11.2%) (n = 423). The mean loss in the lower three quartiles of psoas major area was − 2.3 ± 7.1% (range − 13.0 to 21.1%) (n = 1267), and paraspinal muscle area loss was 0.1 ± 7.1% (range − 11.2 to 20.9%) (n = 1267). The results of a multivariate analysis evaluating risk factors of both psoas major and paraspinal muscle area loss are presented in Table 2. Only aging [≥ 50 s (odds ratio [OR], 1.72; 95% confidence interval [CI] 1.05–2.84; *p* = 0.03) and ≥ 60 s (OR, 2.67; 95% CI 1.55–4.60; *p* < 0.001)] was a risk factor for decreases in the psoas major area. Age ≥ 60 years (OR, 2.05; 95% CI 1.24–3.39; *p* = 0.005), BMI ≥ 25 kg/m^2^ (OR, 1.32; 95% CI 1.01–1.73; *p* = 0.04), and visceral fat ≥ 100 cm^2^ (OR, 1.61; 95% CI 1.20–2.15; *p* = 0.001) were risk factors for decreases in paraspinal muscle area, while physical activity ≥ 900 kcal/week (OR, 0.68; 95% CI 0.50–0.94; *p* = 0.02) reduced paraspinal muscle area loss in male participants.

**Table 2:** Multivariate analysis for predicting changes in the psoas major and paraspinal muscle area.

	Risk factors		Score assigned	Psoas major			Paraspinal muscle		
				OR	95% CI	*p* value	OR	95% CI	*p* value
All generations	Age	30 s	0	Reference	–	–	Reference	–	–
		40 s	1	1.52	0.90–2.56	0.12	0.76	0.46–1.24	0.27
		50 s	2	1.72	1.05–2.84	0.03	1.26	0.80–1.98	0.33
		60 s	3	2.67	1.55–4.60	< 0.001	2.05	1.24–3.39	0.005
	Alcohol	−	0	Reference	–	–	Reference	–	–
		+	1	0.91	0.70–1.19	0.49	0.95	0.72–1.24	0.69
	Smoking	−	0	Reference	–	–	Reference	–	–
		+	1	1.01	0.81–1.27	0.92	0.84	0.67–1.06	0.15
	BMI (kg/m^2^)	< 25.0	0	Reference	–	–	Reference	–	–
		≥ 25.0	1	1.21	0.92–1.58	0.17	1.32	1.01–1.73	0.04
	Body fat (%)	< 25.0	0	Reference	–	–	Reference	–	–
		≥ 25.0	1	0.87	0.64–1.17	0.34	1.05	0.78–1.40	0.75
	Visceral fat (cm^2^)	< 100.0	0	Reference	–	–	Reference	–	–
		≥ 100.0	1	1.24	0.94–1.63	0.13	1.61	1.20–2.15	0.001
	Physical activity (kcal/week)	< 275.0	0	Reference	–	–	Reference	–	–
		< 510.0	1	1.19	0.87–1.65	0.28	0.90	0.66–1.24	0.53
		< 900.0	2	1.06	0.78–1.46	0.70	0.81	0.59–1.10	0.17
		≥ 900.0	3	1.19	0.86–1.63	0.29	0.68	0.50–0.94	0.02

## Discussion

We longitudinally investigated changes in psoas major and paraspinal muscle area of healthy individuals and determined the risk factors for psoas major and paraspinal muscle loss according to age group and sex. Both psoas and paraspinal muscle areas significantly decreased after a 10-year period for all age groups and sexes in individuals starting in their 30 s. The rate of psoas major and paraspinal muscle area loss increased gradually with age except for psoas muscle area loss in female participants. A regression analysis revealed that only age ≥ 50 years was a risk factor for decreases in psoas major area. Age ≥ 60 years, BMI ≥ 25 kg/m^2^, and visceral fat ≥ 100 cm^2^ were risk factors for decreases in paraspinal muscle area, while physical activity ≥ 900 kcal/week reduced paraspinal muscle area loss in male participants.

Age-related muscle change in paraspinal muscle or skeletal muscle has been examined in cross-sectional studies. Sasaki et al. measured the paraspinal muscle area of 796 Japanese participants (mean age = 63.5 years, categorized as < 50, 50–59, 60–69, 70–79, and ≥ 80 years old) in a cross-sectional study and revealed that muscle area significantly decreased in individuals in their 50 s compared to those aged < 50 years^15^. Another cross-sectional study examined the influence of age and whole-body skeletal muscle mass in 468 participants (age range 18–88 years) using magnetic resonance imaging and found that skeletal muscle mass gradually increased until individuals were in their 30 s and started to decrease around ages of 45–50 years^16^. However, the present longitudinal study is the first to provide evidence of a noticeable decrease in muscle area occurring in individuals in their 30 s since it included healthy individuals from multiple age groups to calculate muscle area loss.

Although previous longitudinal studies have reported significant changes in the cross-sectional muscle area, most investigated the lower extremity muscle area and included older individuals. In a study of 1880 older adults, Goodpaster et al. reported an annual reduction of approximately 1% in the lean leg area^17^. Delmonico et al. reported changes in the mid-thigh muscle area in individuals in their 70 s over a 5-year period and found a 3–5% decrease^18^. The rate of muscle area loss per year in the present study was lower than in previous studies, and this is likely due to study differences in the measured muscle and age groups targeted. In the longitudinal study for paraspinal muscle, Mäki et al. longitudinally investigated 298 participants (mean age, 21.2 years; range 19–22) with follow-up scans conducted at a mean age of 30.6 years and found that the paraspinal muscle area, except for the area of the psoas major, increased in the young adult population (20–30 years of age)^19^. Therefore, our results add to the aforementioned evidence that the psoas major and paraspinal muscle area, except for the area of the psoas major, increases in individuals from their 20 s to 30 s, and both psoas major and paraspinal muscle areas decrease after that.

Our research indicated that alcohol and smoking were not leading risk factors for a decrease in psoas major and paraspinal muscle areas. A previous study found that skeletal muscle autophagy increased with alcohol consumption^20^. However, another study on alcohol consumption effects reported no sarcopenia correlation^21^. Our results support this as we found no association of alcohol consumption with psoas major and paraspinal muscle areas.

Previous studies have reported an association between physical activity and muscle strength in older people^22,23^. In a 5-year longitudinal analysis, Rantanen et al. demonstrated that maintaining activity levels prevents muscle strength decline with age^24^. Our study confirms these trends and adds evidence to the amount of physical activity required. Hao et al. reported an association between the skeletal muscle mass index (SMMI) and physical activity for 640 adolescents in short-term outcomes and suggested that SMMI was positively associated with physical activity^25^. However, there is limited knowledge regarding the association between skeletal muscle mass changes and the amount of physical activity for individuals aged 30–60. Our study indicates that exercise burning 900 kcal/week, including daily movement such as commuting on foot, attenuates paraspinal muscle area loss in male participants. This translates to walking at 4.0 km/h for 300 min/week for individuals weighing 60 kg. Based on our findings, we recommend that male participants walk more than 45 min daily.

Fortin et al. provided evidence that greater multifidus and erector spinae fatty infiltration at L5/S1 was associated with low back pain at the 1-year follow-up^26^. On the other hand, their 15-year longitudinal magnetic resonance imaging study concluded that the level of physical activity at work and leisure and low back pain were not associated with changes in paraspinal muscle morphology^27^. Additionally, Gelhorn et al.^28^ found that the cross-sectional area of paraspinal muscles could not predict low back pain at the 6- or 12-month follow-up in older adults with spinal degeneration. Hebert et al.^29^ explored the cross-sectional relationships between lumbar multifidus intramuscular adipose tissue infiltration and low back pain, reporting that such infiltration was consistently associated with low back pain at the age of 40 years, although these associations were no longer present at the ages of 45 or 50 years. Ito et al.^30^ investigated the association between the cross-sectional area of the trunk muscles (the erector spinae and multifidus) and falls in older patients with lumbar spinal stenosis, demonstrating that the cross-sectional area of the L4/5 multifidus was related to fall risk. Furthermore, other studies have reported that the sagittal alignment of spine is associated with falls. Ishikawa et al.^31^ reported that a lumbar lordosis angle of 3 degrees or less was associated with falls in older individuals. Additionally, one report noted that the horizontal distance between the C7 plumb line and the center of the ankle is associated with the risk of falls^32^. Given the lack of functional data in the present study, further studies are required to determine risk factors influencing function, low back pain, and fall risk.

The longitudinal study design and large sample size of healthy individuals are two strengths of the present study. Moreover, a multivariate analysis with variables from comprehensive health data allowed for robust analysis. However, this study has some limitations. First, participants were not randomly selected from the general Japanese population since screenings were conducted for employees of corporations in Hitachi city. Additionally, there is a possibility that the visceral fat site as measured by CT revealed differences between baseline and 10-year follow-up values as CT imaging was done at the umbilicus level; however, we accounted for this by excluding individuals whose CT scans were not between the L4 upper edge and L4/5 disc area. Third, there is a clear lack of information regarding the history of low back pain in the selected individuals. However, participants with low back pain that resulted in leaves of absence were not included in this study, as measurements were obtained at annual health check-ups for workers. Fourth, the areas of the multifidus and erector spinae muscles were not measured separately since we used CT, even though MRI is the gold-standard method for assessing paraspinal muscle area and composition. Fifth, since this study focused on changes in muscle area only, future studies should examine changes in fatty infiltration for each muscle. Additionally, the reliability of the software used in this study remains to be reported. Finally, this study did not assess the inter- and intra-observer reliability of muscle area measurements even though measurements were manually minute-adjusted in the only case when the scan seemed to be in correct.

In conclusion, the present findings demonstrate that psoas major and paraspinal muscle areas significantly decreased over a 10-year period for all age groups, starting in the 30 s. Aging was a risk factor for decreases in the psoas major area. Additionally, aging, obesity, and visceral obesity were risk factors for decreases in paraspinal muscle area. However, physical activity ≥ 900 kcal/week reduced paraspinal muscle area loss in male participants. This finding indicates that walking for > 45 min daily (Calories = METs (walking: 3.0) × duration of time (hr) × weight (60 kg) × 1.05) can reduce paraspinal muscle loss, which may in turn decrease the risk of falls, low back pain, and aid sarcopenia.

## Methods

### Study participants

This study was approved by the Ethics Committee of Medical Research of the University of Occupational and Environmental Health Institutional Review Board in accordance with the Declaration of Helsinki 2013. Informed consent was obtained from all participants, and all experiments were performed according to relevant guidelines and regulations.

Individuals who underwent a low-dose CT screening of the abdominal visceral fat area during annual health check-ups from April 2004 to March 2006 at the Hitachi Health Care Center were recruited for this study. For individuals screened twice within the period, the first set of data was used. CT measurements of visceral fat area were performed as previously described^33–35^. To minimize radiation exposure, single-slice imaging was performed at the level of the umbilicus in the supine position using a Radix Turbo CT scanner (Hitachi Medico, Tokyo, Japan). Imaging conditions were set at 120 kV and 50 mA, with a slice thickness of 5 mm. For the annual examinations, we standardized imaging at the level of the umbilicus and the most of this level was between L4 upper edge and L4/5 disc level^36,37^. L4 upper edge and L4/5 disc level was selected for the analysis because these levels have been used in previous studies^9,15,38^. We selected CT slices that included the L4 upper edge and L4/5 disc level while referring to the characteristics of the iliac bone; run of the aorta; and the shapes of the quadratus, psoas, and vertebra. These are totally different from those at the level of L3/4 or L5. Any disagreement was addressed via discussion between the two spine surgeons. Individuals whose cross-sectional CT images did not include the area between the L4 upper edge and the L4/5 disc, as well as those with history of lumbar surgery, were excluded. Individuals who did not undergo CT screening 10 years after their initial screening and whose CT images did not include the area between the L4 upper edge and L4/5 disc at the 10-year screening were excluded.

### Examinations

#### Self-administered demographic and health questionnaire

Individuals completed a self-administered questionnaire with lifestyle-related questions including occupation, work posture, smoking habits, alcohol consumption, family history, medical history, physical activity, and health-related QOL. For this study, data on the frequency and current or past use of cigarettes and alcohol consumption were collected. Those who never or occasionally drank alcohol were considered non-drinkers, and those who never smoked or smoked in the past were considered non-smokers.

#### Physical activity

Physical activity was calculated according to previous reports^39^. Information on physical activity was obtained from the questionnaire. When preferred activities were not listed, activities of similar exertion levels were selected. Of the 20 types of exercise listed, “other” was not used. Metabolic equivalents (METs) of each activity based on physical activity guidelines were assigned (if the MET value was not on the list, a related value was used)^40^. Of the 19 exercises, 13 [work and commuting, walking, swimming, golf practice, golf, baseball, softball, cycling, table tennis, badminton, strength training, light jogging (approximately 6 min/km), jogging, soccer, tennis, aerobics, and jump rope] were active activities (> 6 METs), and weekly and hourly METs were calculated. Walking time for commuting was self-reported. Calories burned during commuting were calculated by multiplying the daily walking time by five (assuming two days off a week). Total physical activity was calculated from the calories burned for regular exercise during leisure plus those for commuting.

#### Anthropometric measurements

Anthropometric measurements were performed. BMI was calculated by dividing weight (kg) by the square of height (m^2^). The percentage of body fat was measured using a bioelectrical impedance analysis where fat mass was divided by total mass and multiplied by 100. The visceral and subcutaneous fat areas at the umbilical level were measured using a CT scanner (Radix turbo; Hitachi Medico, Tokyo, Japan) and automatically estimated using fatPointer software (Hitachi Medico, Tokyo, Japan) as previously described^33–35^.

#### Psoas and paraspinal muscle area

**Figure 3:**
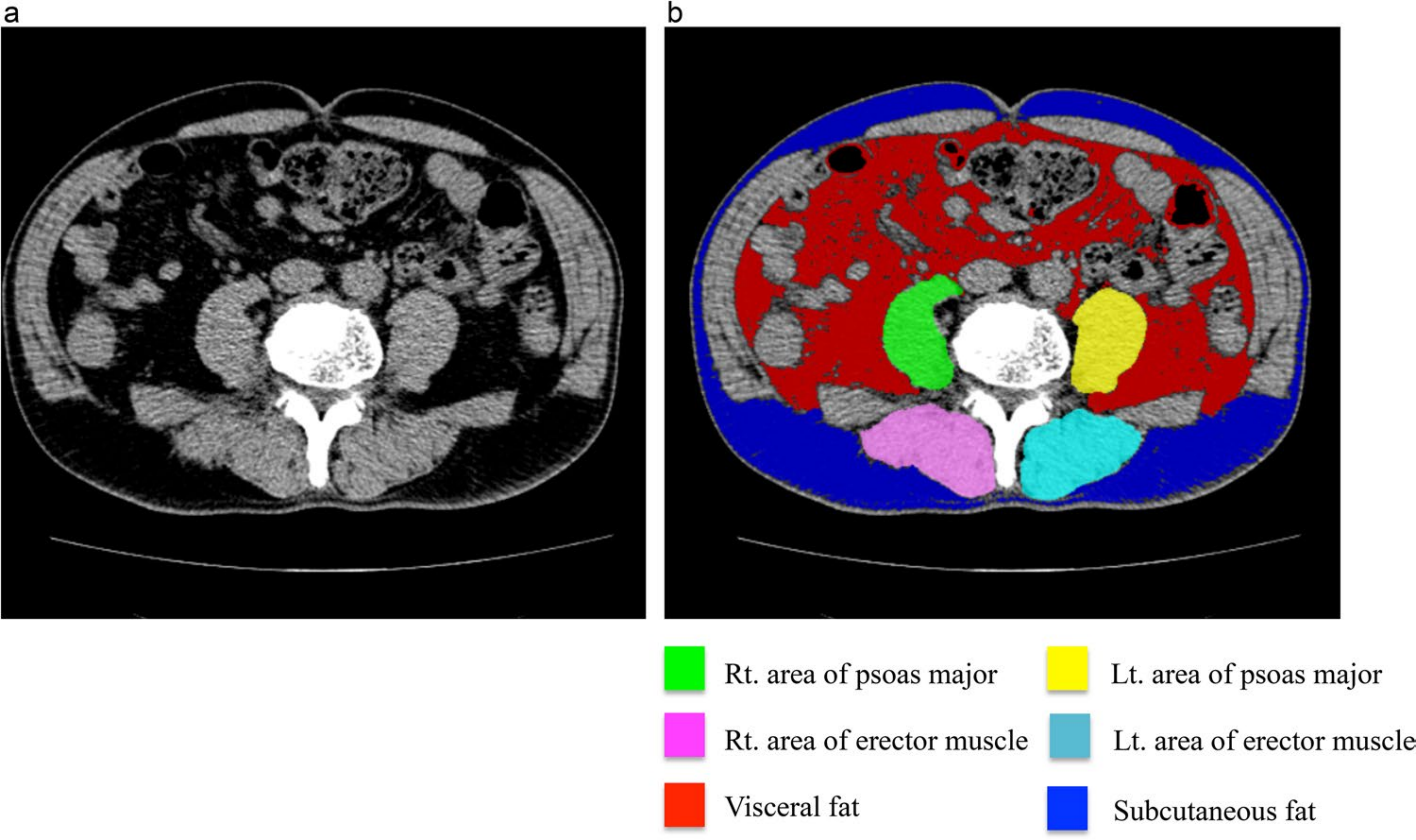
Representative view with musclePointer software. (**a**) An original image at the level of the umbilicus as observed using computed tomography. (**b**) Software calculated the area of each muscle automatically.

Psoas major and paraspinal muscle areas were assessed by a retrospective examination of CT scans utilizing musclePointer software (HITACHI Ltd., Tokyo, Japan). This software was developed as an evolution of fatPointer, which was used to measure cross-sectional visceral and subcutaneous fat areas and has been cited in some previous studies^36,37,41^. An engineer among the co-authors of the present study developed this automated software. CT images were divided by the threshold value of − 400 Hounsfield units (HU) into two cross-sectional areas: air and other tissues. The skin area was removed, and the areas of other tissues left in CT images were divided by the threshold value of -50 HU into two cross-sectional areas: fat and bone, muscle. Software automatically scanned each muscle (bilateral psoas major and paraspinal muscle). The paraspinal muscle group contains the multifidus and the erector spinae. The borders between the multifidus and erector spinae muscles are often difficult to distinguish, as mentioned in previous studies^38,42^ Therefore, the multifidus and erector spinae muscles were measured together and considered as the paraspinal muscle. Six orthopedic surgeons performed pilot measurements using several dozen slices to control for inter-observer variability in the interpretation of the images. Measurements were manually minute-adjusted by at least two orthopedic surgeons in the only case when the scan seemed to be incorrect. Each area was calculated, and data were entered into an Excel spreadsheet (Fig. 3).

### Statistical analysis

For descriptive variables, individuals were grouped by age in 10-year increments and by sex. Demographic and clinical characteristics are summarized as means ± standard deviations. Muscle areas between baseline and the 10-year follow-up were compared using a paired t-test. A one-way analysis of variance was used to compare baseline demographic variables, muscle area, and fat area composition according to age group for each sex. Given the small number of female participants, a logistic regression analysis was performed for male participants only. Four groups were created according to quartiles of muscle area loss. The group with the lower three quartiles (bottom 75%) was designated as the reference group. Covariates were dichotomized with reference groups of non-drinkers, non-smokers, BMI < 25 kg/m^2^, body fat < 25%, and visceral fat < 100 cm^2^, except for physical activity, which was split into quartiles. Associations between muscle area loss and potential risk factors were assessed via a multivariable logistic regression analysis, with muscle area loss dichotomized into the highest quartile of muscle area lost (top 25%) versus the lower three quartiles (bottom 75%). ORs and 95% CIs were calculated using the reference group. All statistical analyses were performed using STATA/IC 14 (StataCorp, College Station, TX, USA). The level of significance was set at a *p* value < 0.05.

## Data Availability

The data are not available for public access because of patient privacy concerns, but are available from the corresponding author on reasonable request.
